# 

**Published:** 1845-01

**Authors:** 


### INDEX.

Abdomen, punctured wound of, 643
Abercrombie's case of hæmorrhage of the liver, 45
Abercrombie, Dr., death of, 70
Abner Rogers, trial of, 124
Abnormal position of the kidneys, Dr. Diver on, 714
Accidents and poisons, Rivers on, 280
Accidental productions, nature and developments of, 636
Acclimating fever of Liberia, 506
Aconite, death from, 641
Acute rheumatism complicated by amnesia, 61
Action of lead in distilled and river water, 362
Action of oils on animal economy, 380
Adulteration of veratria with lime, 626
Africans, exemption of, from insanity, 178
Air, compressed, effects of, 335
" passages, sojourn of a cherry stone nine months in the, 385
Albany Medical College, annual circular of, 620
Alcohol, intoxicating doses of, in traumatic tetanus, 319
American Medical Biography, 110
" Journal of Insanity, 114, 500
Amnesia, complicating acute rheumatism, 61
Amussat's operation for artificial anus, 159
Analysis of blood in a case of lead colic, 57
Anatomy of the thymus gland, Sir A. Cooper on, 109
Anatomy, Pathological, Hope's, 411
" &c. of sexual organs, Guthrie on, 486
" and diseases of the breast, Sir A. Cooper on, 616
Anatomical remembrancer, 731
Anecdota Sydenhamiana, 613
Anencephalus, human, history of, 435
Aneurismal tumour of bone, 375
Aneurism, observations on, 347
" dissecting, of thoracic aorta, peculiar form of, 385
Animalcules, spermatic, Prof. Berutti on, 508
Annual announcements of medical lectures, 551
Annual circulars of medical colleges, 620
" announcement of Jefferson Medical College, 620
" announcement of Rush Medical College, 622
Annual circular of Washington University of Baltimore, 620
" election of Philadelphia Medical Society, 33
Anterior obliquity of uterus, 573
Antrum tubæ of Rœderer, 580
Aorta, abdominal, aneurism of, 193
" thoracic, peculiar form of dissecting aneurism of, 385
Ardent spirits, death from excessive use of, 70
Arsenic, process for detection of, 317
" blood as an antidote to, 453
Artificial anus, Mütter on establishment of, 156
Artificial anus, Amussat's operation for 158
" Ashmead's operation for, 159
Ascites, 595
Ashmead's operation for artificial anus, 159
Ashwell on diseases of women, 225
Asylum, Lunatic, Rhode Island, 135
" first in the U. S., 135
" Ohio, Sixth Annual
" New York State, 282
" Kentucky, 287
" Williamsburg, 286
" Insane, Bloomingdale, 284
Asylums, Lunatic, of Paris, 426
Asphyxia, Solar, 526
Asthma, 96
Assistant surgeons qualified for promotion, &c., 34
Atrophy, mechanism or nature of, 47
Auto-plastic operation for cure of ectropium, 252
Autoplasty, 322
" re-establishment of nervous influence after, 377
Augusta, (Me.) report of insane hospital of, 220

Bailey's, Dr. O., new views on epidemic dysentery, 583
Bamfield on curvatures and diseases of the spine, 184
Barth's manual of auscultation and percussion, 730
Beck's manual of chemistry, 29
Bed sores, treatment of, 46
Bell, Sir Chas., on anatomy and physiology of expression, 179
Bell, Dr. John, on practice of physic, 26
" case of congenital malformation of heart, 553
Belladonna in hooping cough, 235

Bibliographical notices, 26, 103, 172
212, 273, 347, 404, 476, 541, 652, 716
Biliary secretion, disorders of, 205
Bigelow's orthopædic surgery, 415
Bi-partite vagina, with rupture of septum and natural delivery, 68
Birch tar, remarkable effects of, 579
Birth of four children, 435
Bird, Dr., on urinary deposites, &c., 723
Blackmore on puerperal fever, 292
Blindness, sudden, case of, 200
Bloomingdale Asylum, 233
Blisters for children, 237
Blood as an antidote to arsenic, 453
" Calvert Holland on, 356
" differences in composition of, in male and female, 507
" formation of buffy coat of, 246
" mass of, in relation to the weight of the body, 381
" modification of, during inflammation, 382, 644
Board of Naval Surgeons, 34
Bone, pulsating tumour of, 306
" aneurismal tumour of, 375
Brain, ramollissement of, 338
Breasts, enlargement of, in a male, 258
Brigham on mental cultivation, &c., 230
British and Foreign Medical Review, 116
Bronchorrhœa, 536
Bryan, Dr. J., on the prevailing influenza, 76
Bryan, Dr. J., on removal of foreign bodies from the ear, 202
Buffy coat of blood, formation of, 246
Buffalo Medical Journal, 418
Burnett, Sir William's patent, 437

Caloric, Dr. S. Metcalf on, 451
Caloricity, febrile, Dr. Dowler on, 359
455, 519, 624,
Cancer, microscopic texture of, 555
Cancerous communication between the stomach and colon, 201
Cannabis indica in chorea, 568
Cap and gown, the, 387
Carbonaceous matter, deposit of, in the tissue of the lungs, 703
Case of abscess in groin, symptoms of hernia and discharge of lumbrici, 448
" of thoracic disease, 18
" of ulceration of the duodenum 50
" of disease of bones and joints, of lower extremities, &c., 62
" of bi-partite vagina, with rupture of septum and natural delivery, 68
" of death from excessive use of ardent spirits, with appearances simulating irritant poisoning on dissection, 70
Case in which sixteen sewing needles and four pins were extracted from the wrist, by H. G. Spencer, 146
" of copper coloured syphilitic eruption affecting the conjunctiva, 198
" of communication between the ventricles of the heart, 261
" of procidentia uteri during labor, 367
" of hydrophobia, 423
" of purpura hæmorrhagica, 438
" Turnbull's, of anasarca and ascites from pressure of tumour, 592
" Dr. Cunningham's, of twins, one being black and the other white, 647
" Dr. Dock's, of congenital cataract of both eyes, 651
" of ulcerated stomach causing death by detachment from peritoneal lining of abdomen, 699
" Dr. Parker's, of placenta prævia, 700
Cases, surgical, Mr. Symes', 309
Caoutchouc as a remedy for tooth ache, 302
Catalepsy with convulsions of right arm, Irby's case of, 11
Causes of plague, 696
Caustics, M. Cazenave on, 443
" fluid, 444
Cautery, actual, in gangrene of the mouth, 510
Cellular tissue, induration of, 268
Cervix uteri, inflammation, &c. of, 570
Cervic scapulæ, fractures of, 136
Chancres, treatment of, 54
" prognosis of, 251
Cherokee Indians and Africans, exemption from insanity, 135
Cherry kernels, poisoning by, 425
" stone nine months in the air passages, 385
Children, blisters for, 237
" convulsions of new born, 435
" signs of auscultation in, M. Trousseau on, 49
Chlorosis, Corneliani on the cause and treatment of, 443
Christison on poisons, 281
Chorea, 396
" treatment of, by cannabis indica, 568
" treatment of, by strychnia, 582
Chylopoietic system, diseases of, 685
Circulatory system, " 683
Classes, clinical, at Blockley Hospital, 190
" medical, 190, 665
Clinical medicine, Canstatt's manual of 615
Clinical medicine and surgery at Philadelphia, 550

Clinics, 16, 22, 90, 162, 205, 268, 338, 395, 468, 534, 595
Clinical instruction, 733
Cold, recovery from apparent death by, 579
" blooded animals, possibility of inflammation in, 386
Colles' lectures on surgery, 498
Colloid tumour in cavity of cranium, 242
Colombat on diseases of females, 225
" " and hygiene of the voice, 357
Colon, unusual form of intussusception of, 303
Compressed air, effects of, 378
Concretion, intestinal, 193
Condie's oration before the Philadelphia Medical Society, 181
Congenital deficiency of sternum, 60
" hernia strangulated in a child, 513
" opacity of the cornea, 574
Conium in painful diseases, 35
Contributions to therapeutics, 35
" the history of malaria, 144
" general surgery, 389
Constitutions, Voigt's observations on, 519
Convention, national, of physicians, 419
Convulsions, salaam, 399
" of new born children, 435
Cookery, modern, by Eliza Acton, 611
Cooper, Sir A., on the testis and thymus gland, 109
" on anatomy and diseases of the breast, 616
" Samuel, on first lines of theory and practice of surgery, 113
Copeland's dictionary of practical medicine, 89, 233, 416, 499,
Copper and zinc, toxical effects of, 378
Cornea, ulcers of the, 555
" congenital opacity of, 574
Corpora lutea, nature of, 384
Correspondents, to, 552
" and publishers, to, 233
Coup de soliel, 626
Croton oil in nævi materni, 46
Croup, tracheotomy in, 45
Cranio malacia, 562
Çrystalline lens, reproduction of, 46
Cunningham's, Dr. H., case of twins, 647
Curvatures and diseases of spine, Bamfield on, 184
Cyclopædia of practical medicine, 30, 189

Damascus, hospital at, 237
Death from an over-dose of nitrate of potassa, 67
" from excessive use of ardent spirits, &c., 70
" from drowning, 168
Death from aconite, 641
" apparent, from cold, 579
" of Dr. Abercrombie, 70
" of Dr. John Houston, 646
" of M. Ribes, 387
" of M. Olivier (d'Angiers,) 388
" of Prof. Graham of Edinburgh, 646
" of Prof. Richardson, 623
" of a surgeon from prussic acid, 258
" sudden, from spontaneous rupture of the stomach, 695
" sudden, cause in the brain, 630
" " with congestion of the right heart and lung, 631
" " result of syncope, 634
" " " epilepsy, 635
De l'identite du typhus et de la fievre typhoide, par E. S. Gaultier, 172
Delivery, premature, cases of, 252
Dental surgery, Harris' principles and practice of, 289
Deposit of carbonaceous matter in the lungs, 703
Deschamps' researches on ovum and corpus luteum, 250
Diagnosis of hepatitis and hepatalgia, 366
Dickson's essays on pathology and therapeutics, 716
Dictionary of medicine, by Adelon Beclard, &c., 212
" of practical medicine, Copeland's, 189, 233, 416, 499
" of terms, Hoblyn's, 659
Differences of blood in male and female, 507
Discoloration from nitrate or silver, 400
Disease regarded as a false or spurious organization, 513
Diseases, chronic, cutaneous, 468
" of the circulating system, 683
" " chylopoietic " 685
" " genito-urinary " 687
" " nervous " 669
" " respiratory " 675
" " " organs, Williams and Clymer on, 103
" " skin, Worcester on, 350
" of children, Stewart on, 663
" scrofulous, Lugol on, 359
" of uncertain seat, 690
" zymotic, 666
Disorders of the biliary secretion, 205
Dislocation of shoulder of seven weeks standing, 309, 311
" " of four weeks standing, 312
" of hip of five weeks standing, 312
" of thigh upwards and backwards, 312
Dispensatory of the U. S. of America, 416

Dixon's practical treatise on diseases of the sexual organs, 730
Dowler on febrile caloricity, 359, 455, 519, 624
Domestic management of the sick-room, Thompson's, 662
Drake's introductory, 188
Dracunculus or Guinea worm, case of, 511
Drowning death from, 168
Dropsy, ovarian, discharging itself by the umbilicus, Squibb's case of, 457
Dropsy of ovum, Liggett's case of, 529
" which follows scarlatina, 567
Duodenum, malformation of, 448
Dunglison's clinical lectures, 16, 90, 162 205, 268, 395, 468, 534, 595
Dysentery, epidemic, Dr. Bailey's new views and cases of, 583

Ectropium, autoplastic operation for, 252
Editorial remarks, 32, 114, 190, 233, 290, 417, 500, 550, 618, 664, 732
Electricity, novel application of, to surgery, 441
Elementary and practical treatise on internal pathology, by A. Grisolle, 212
Elementary treatise on internal pathology, by MM. Hardy and Behier, 212
Elements of chemistry, Fownes', 549
" materia medica and therapeutics, Harrison's, 652
Elliot's, W. H., contributions to surgery, 389
Embalmment by injection, 701
Endermic use of tobacco, Diver on, 527
Endocarditis complicated with valvular disease, 596
Enlargement of breasts in a male with atrophy of genitals, 258
Eppes' case of spasm of the glottis, 264
Epizootic in Germany, 377
Epilepsy, autopsy in a case of, resulting from an injury of the head, 263, 472
Epilepsy causing sudden death, 635
Erysipelas, erratic, 197
Escape of a tenia solium through the umbilicus, 96
Esquirol on insanity, 476
Ether, sulphuric, death from the inhalation of, 502
Experiments on the action of oils on the animal economy, 380
" on the mass of blood relative to the weight of the body, 697
" by Dr. F. G. Smith on the hearts of the sturgeon, snapping turtle and frog, 393
Expression, Sir C. Bell on, 179
Extensive scrofulous disease, 192
Extirpation of spleen and thyroid gland, effects of, 250
Extirpation of lacrymal gland for the cure of fistula lacrymalis, 252
Extra-uterine pregnancy, removal of child by Cæsarean operation; Dr. McCulloch's case, 701

Febrile caloricity, Dr. B. Dowler on, 359, 455, 519, 624
" periodicity as influenced by sulphate of quinia, Prof. T. D. Mitchell on, 386
Female, case of lithotomy in, 369
" difference of blood in male and, 507
Females, Colombat on diseases of, 225
Fever, intermittent, 90
" acclimating, of Liberia, 506
Fibrous tumour of inferior maxilla, 316
Fingers, remarkable hypertrophy of, in a girl, 447
First lines of theory and practice of surgery, by Samuel Cooper, 113
First lunatic asylum in the United States, 135
Fish hook removed from œsophagus without an operation, 254
Fistula lachrymalis, cure by extirpation of lachrymal gland of, 252
Fistulous communication between ileum and urinary bladder, with symptoms of stone, 43
Fluid, nervous, 35
Foramen ovale open in a woman 90 years of age, 401
Foreign bodies in the system, 259
" body in the rectum, 436
Forensic medicine, Guy's principles of, 277
Fownes' chemistry, 549
Fractures of the cervix scapulæ, 136
Frog, experiments on the heart of a, 393
Frontal and temporal neuralgia, 582
Fungus hæmatodes, 136
" of inferior maxillary, Harrington's case of, 461
" of synovial capsule of knee treated by compression, 68
Gangrene of the mouth, actual cautery successfully employed in, 510
Gastrorrhœa, 536
Gastritis, chronic, 534
Gaultier, de l'identite du typhus et de la fievre typhoide, 172
Genitals, malformation of, 55
Genito-urinary system, diseases of, 687
Generation, spontaneous, &c., Prof. Berutti on, 508
Gerdy's case of fungus of synovial membrane of knee successfully treated by compression, 68

Glanders, fatal case of, 47
" case of, in the human subject, 758
Glottis, remarkable case of spasm of the, 264
Goule, trial of, for murder, 644
Goitre in Switzerland, 760
Graham, Prof., death of, 646
Griscom on the sanatory condition of the labouring classes of New York, 230
Grisolle's elementary and practical treatise on internal pathology, 212
Groce's, Dr. B. W., case of lithotomy in a female, 369
Groin, Howell's case of abscess in, with discharge of lumbrici, 448
Guinea worm, case of, 511
Guthrie on sexual organs, 486
Guy's principles of forensic medicine, 277

Hæmorrhage from the liver, case of, 45
" unavoidable, Prof. Simpson on, 367
" from leech bites, method of arresting, 378
Half-yearly abstract, Ranking's, 617
Hare-lip in the negro, 505
Harden's, Dr. J. B., case of procidentia uteri during labour, 367
Hardy and Behier's elementary treatise on internal pathology, 212
Harris' dental surgery, 289
Harris and Meigs' Velpeau, 607
Harrison's materia medica and therapeutics, 651
Harrington's case of fungus of the inferior maxillary,. 461
Haywood's case of lithotomy in a child three years old, 9
Head, wound of, with loss of substance of the brain, 696
Health of Philadelphia, 290, 664
Heart, enlargement of, complicated with valvular disease, 257
" communication between ventricles of, 261
" of the sturgeon, Dr. F. G. Smith's experiments on, 393
" congenital malformation of, 553
" great " " 757
" treatment of hypertrophy of, 579
Hemiplegia, 257, 341
Hepatitis and hepatalgia, diagnosis of, 366
Hereditary transmission of insanity, 566
Hernia, congenital, strangulated in a child, 513
Hints on the reorganization of the navy 188
Hobnailed liver, case of, 191
Hoblyn's dictionary of terms, 659
Holmes' contributions to the history of malaria, 144
Holland, Calvert, on the moving powers of the blood, 356
Hooping cough, 235
Hope's morbid anatomy, 411
Horner's medical topography, 111
Hospital, Philadelphia, 16
" " clinical class at, 190
" " clinics at, 33, 90, 162, 205, 268, 338, 395, 468, 534, 595
Hospital, Pennsylvania, 22
" " for insane, report of, for 1844, 220
" Wills, 22
" at Damascus, 237
Houston, Dr. John, death of, 646
Human anencephalus, history of, 435
Hunt on relaxed rectum, 247
Hydrocele treatment by ioduretted injections, 69
Hydrocyanic acid, death of a surgeon from, 258
Hydrophobia, case of, 423
Hygiene, Levy on, 541
Hypertrophy, remarkable, of fingers in a girl, 447
Hypertrophy of heart, treatment of, 579
" of spleen, remarkable, 580

Ileum, perforation of, 136
Improved life preserver, 756
Inferior cava, spontaneous rupture of, 57
" maxillary, malignant tumour of, 315
" " fungus of, 461
Infants, jaundice of, 211
Inflammation, modification of blood in, 382
" possibility of, in cold-blooded animals, 386
" &c. of cervix uteri, 570
Influenza, Dr. Bryan on, 76
" among horses, 305
Intestinal concretion, 193
" canal, stricture of, 470
Intestines, paralysis of, 301
" large obstruction of, Dr. Evans' case of, 371
" small, intussusception of, 379
Intussusception of colon, unusual form of, 303
" of small intestine case of, 379
Intra-thoracic solid tumours, 626
Insanity, American Journal of, 114, 500
" Cherokees and Africans, exemption from, 135
" Esquirol's treatise on, 476
" hereditary transmission of, 566
Iodide of potassium in pneumonia, further remarks on, by Dr. Upshur, 323
Irby's case of catalepsy, &c., 11

John, Dr., on disease as a false or spurious organization, 513
Jaundice of the infant, 211
Jefferson Medical College, annual announcement of, 620
Jobert's rare case of milk cyst in the breast, 58
Journal, American of Insanity, 114, 500
" Buffalo Medical, 418
" Missouri Med. and Surg., 418
" new medical, in New Orleans, 501
" New Orleans Med. & Surg., 619
" New York, of Medicine, 500
" Pennsylvania, of Prison Discipline, 541
" Southern Med. and Surg., 115
" St. Louis Med. and Surg., 522
Journals, medical, new, 417

Kilpatrick's case of fish-hook removed from the œsophagus without an operation, 254
Kidneys, passage of foreign substances through the, 567

Lachrymal gland, extirpation of, 252
Lamballe, M. Jobert de, 563
Laporte University, (Indiana,) 34
Lardaceous scirrhoma of the lung, involving the first rib, &c., 54
Lead colic, analysis of the blood in a case of, 57
Lead, action of, in distilled and river water, 362
Lectures, Dunglison's, 16, 90, 162, 205, 268, 338, 395, 468, 534, 595
" Watson's, on practice, 602
" Spencer's, on animal heat, 273
" on theory and practice of surgery, Colles', 498
Leech bites, simple method of arresting hæmorrhage from, 378
Lee's introductory, 31
Lefevre's, Sir George, apology for the nerves, 404
Liberia, acclimating fever of, 506
Ligature of a polypus of the bladder, 257
Lithotomy in a child three years old, 9
" in a female, double calculus, 369
" " Dr. Baker's case, 420
Liver, hobnailed case of, 191
" encysted tumour of, 316
" hæmorrhage from, case of, 45
Lugol on scrofula, 354
Lumbrici, discharge of, from an abscess in the groin, 448
Lunatic asylum, Rhode Island, 135
" first, in the U. States, 135
" Ohio, sixth Annual Report of, 220
Lunatic asylums at Paris, 426
Lung, lardaceous scirrhoma of, 54
" deposit of carbonaceous matter in the, 703

Malaria, contribution to the history of, by Dr. Holmes, 144
Malignant tumour of inferior maxilla, 315
Manual of clinical medicine, 615
Malformation, congenital, of the heart, 553
" of the duodenum, 448
" of the genitals, 55
Mechanism or nature of atrophy, 47
Medical classes, 190, 665
" institution of Yale College, 116
" college of South Carolina, 501
" and Chirurgical Society of London, 235
" journals, new, 417
" lectures, annual, announcements of, 551
" lexicon, Reese's, 106
" matters in Prussia, 364
" remembrancer, Shaw's, 499
" student's vade-mecum, 112
" topography of Brazil and Uruguay, Horner's, 111
" Examiner, new series of, 32
Medical Society, London, 235
" London, 72d anniversary of, 387
" New York State, 413
" Philadelphia, 33, 181
" Sheffield, 257
" Vermont 34
" Westminster, 259
Medicine in Siam, 506
Medical classes in Philadelphia, 733
Melancholia puerperalis atonicta, 60
Memoir on uretro-plastie, 640
Mendenhall's medical student's vade mecum, 512
Menstruation and periodic discharge of ova, 112
Mental cultivation and excitement, Brigham on, 230
Mental maladies, Esquirol on, 476
M'Culloch's, Dr. M., case of extra uterine pregnancy, requiring Cæsarean operation, 701
Mesmerism, 233
Metallic mercury, passage of, into the blood, &c., 756
Metcalf, Dr. Samuel L., on caloric, 541
Microscopic texture of cancer, 555
" investigations, utility of, 572
Midwifery Velpeau's, 607
Milk cyst in the breast, rare case of, 58
Miller, Dr. J., case of fatal phrenitis from inhalation of sulphuric ether, 502

Miller's, Thos., case of coloid tumour in cavity of cranium, 242
Miller's principles of surgery, 228
Missouri medical and surgical journal, 418
Mitchell's, Prof. J. K., cases of scarlatina, 7
Mitchell's, Prof. J. K., case of racemiferous hydatids of the uterus, 73
Mitchell, Prof. T. D., on sulphate of quinine, 386
Modern cookery, by Eliza Acton, 611
Mollites ossium, Solly on, 44
Monro's case of disease of bones and joints of the lower extremity, 62
Monstrosity, case of, 695
Mortality attending the operation of tying the large arteries, 383
Mott's Velpeau's surgery, 175
Mounting preparations under thin glass, 575
Murder, quack medicines, &c., 115
" trial of Goule for, 644
Mütter on strictures of the rectum, 77, 148

Nævi materni cure by croton oil, 46
National convention of physicians, 419
Nature and development of accidental productions, 636
Naval surgeons, board of, 34
" " assistants admitted to the U. S. navy, 34
Navy, hints on the re-organization of, 188
" and Transylvania University, 419
Negro, hare lip in, 505
Nervous fluid, 135
" action, re-establishment of, after autoplastic operations, 377
Nervous system, diseases of, 669
Neuralgia, frontal and temporal, 582
New elements of operative surgery by Valpeau and Mott, 175
New York medical journals, 417
N. York medical and surgical reporter, 732
New York journal of medicine, &c., 500
New York medical intelligencer, 619
New Orleans, new medical journal in, 501
" physico-medical society of, 564
New medical and surgical journal, 619
Nicol on preserving bodies for dissection, 61
Nitrate of potassa, death from an overdose of, 67
Northern schools and Southern medicine, 137

Observations, hints on relaxed rectum, 247
" on aneurism, 347
" on constitutions, Voigt's, 519
Obstruction of large intestine, ascending colon opened, Evans's case of, 371
Obstruction and obliteration of vena cava, 51
Obituary, 291
Occasional pulsation of the veins, 194
Occlusion of vagina in a pregnant woman, 255
Oesterlen on the passage of metallic mercury into the blood, 55
Ohio lunatic asylum, sixth annual report of, 220
Oils, action of, on the animal economy, 380
Ointments, preparation and preservation of, 518
Oliver, M. d'Angers, death of, 338
On the structure and diseases of the testis, Sir Astley Cooper, 109
On the dressing of wounds by occlusion, 710
Operation, Amussat's, for artificial anus, 158
Operation for excision of the rectum, 154
" auto-plastic, for ectropium, 252
" of tying large arteries, mortality attending,
Open foramen ovale, with pulmonary consumption, in a female ninety eight years of age, 401
Os hyoides, spontaneous expulsion of, 196
Os uteri, simple ulceration of, 449
Ovarian dropsy discharging by the umbilicus, 60
Ovarian dropsy, Squibb's case of, 457
Ova, periodic discharge of, &c., 512
Ovum, Liggett's case of dropsy of, 529
Ox gall, remedial efficacy of, 580

Paralysis of intestines, 301
Paraplegia, 395
Paris, notes of lunatic asylums of, 426
Parker's Cooper's first lines of surgery, 113
Passage of metallic mercury into the blood and other organs of the body, 55
Parisian medical schools, 711
Pathological specimens, 19, 98, 270, 345, 401, 539
Paulus Ægineta, the seven books of, 347
Peacock's case of obstruction and obliteration of vena cava, 51
Peritonitis following examination for uterine polypus, 644
Pennsylvania journal of prison discipline, 541
Pennsylvania hospital, 22
" " for insane, 220
Perforation of the ileum, 136
Philadelphia, college of physicians, transactions of, 704
Philadelphia college of physicians, Condie's oration before, 181

Philadelphia medical society, annual election of, 33
Philadelphia Hospital, 33
" clinics at, 16, 90, 162, 205, 268, 338, 395, 468, 534, 594
" clinical class at, 190
" clinical medicine and surgery in, 550
" health of, 664
Physicians, national convention of, 419
Phrenitis and death from inhalation of sulphuric ether, 502
Placenta Prævia, Parker's case of, 700
Plague, causes of, 696
Pneumonia, iodide of potassium in, 323
Poisons and acccidents, Rivers on, 280
" Christison on, 281
Poisoning by cherry kernels, 425
Polypus of the bladder, ligature in, 257
Prael's case of rupture of the uterus and abdominal parietes, 238
Pregnancy, extra uterine, removal of child by Cæsarean operation, 701
Premature delivery, cases of, 252
Preparations, mounting of, under thin glass, 575
Preparation and preservation of ointments, 518
Preservation of bodies for dissection, 61
Process for the detection of arsenic, 817
Procidentia uteri during labor, case of, 367
Prognosis of chancre, 251
Prospectus, Dr. Greenhill's, 622
Prussia, medical matters in, 364
Puerperal fever, Blackmore's observations on, 292
Pulmonatory fistula consecutive on a scrofulous necrosis, 762
Pulsating tumours of bone, 306
Punctured wound of abdomen, 643
Punctures, subcutaneous, 700
Purgatives, combination of, with copaiba, 251
Purpura hæmorrhagica, case of, 438

Quackery, 501
Quack medicines, 115
Quarantine laws, 564
Quinine, large doses in the treatment of bilious remittent fevers, 749

Raciborski on the nature of corpora lutea, &c., 384
Racemiferous hydatids of the uterus, Dr. J. K. Mitchell's case of, 73
Ramollissement of the brain, 338
Rankin's abstract, 617
" report on the progress of practical medicine, pathology and therapeutics, 666
Rectum, Mütter on strictures of, 77
" simple stricture of, 78
" permanent stricture of, 80
" malignant stricture of, 148
" excision of, 154
" relaxed, Hunt on, 247
" ulceration of the, 403
" foreign body in, 436
Reese's medical lexicon, 106
Re-establishment of nervous action after autoplastic operations, 377
Remarkable case of spasms of the glottis, 264
Remedial efficacy of ox gall, 580
Removal of foreign bodies from the meatus auditorius externus, 202
Removal of part of the base of the tongue, 53
Re-organization of the navy, hints on, 188
Researches on the ovum and corpus luteum, by M. Deschamps, 250
Review, British and Foreign, 116
Report of Augusta (Me.) insane hospital, 220
Report of Bloomingdale asylum, 24th annual, 284
Report of Eastern asylum at Williamsburg (Va.) 286
Report of Kentucky lunatic asylum, 287
" of N. Y. State lunatic asylum, 282
" of Ohio lunatic asylum, 6th annual, 220
" of Pennsylvania hospital for the insane, 220
" of asylum for relief of persons deprived of the use of their reason, 288
" of the yellow fever at Woodville, Miss., by Drs. Valletti and Logan, 117
" of the trial of Abner Rogers, Jr. 124
" French, on vaccination, 560
" first and second, of commissioners for inquiring into the state of large towns and districts, 541
" on the medical department of the University of Penna., 621
" Ranking's, on the progress of practical medicine, pathology and therapeutics, 666
Respiratory system, diseases of, 675
Respiration, jerking, 96, 344
Respiration, phenomena of, 762
Respiratory organs, Williams and Clymer on, 103
Rheumatism, 16, 537
Rhode Island lunatic asylum, 135
Richardson, Prof., death of, 623
Ricord's prognosis of chancre, &c., 251
Rivers on accidents and poisons, 280

Rogers, Abner, trial of, 124
Royal medical and surgical society, 371
Rumination in the human subject, 314
Rupture of the uterus and abdominal parietes, case of, by Dr. Prael, 238
Rupture, spontaneous, of the inferior cava, 57
Rupture, spontaneous, of the stomach, 695
Rush medical college, third annual announcement of, 632

Salaam convulsion, 399
Saltpetre, explosion of, 664
Sanatory condition of the labouring population of New York, 230
Scarlatina, Dr. J. K. Mitchell on the contagiousness of, 7
Scarlatina, the dropsy which follows, 567
School of medicine and surgery, Montreal, 620
Scrofula, Lugol on, 354
Scrofulosis, 162
Section of the tendons of the knee in white swelling of the knee, by M. Ribes, 56
Sewing needles and pins extracted from the wrist, 146
Sexual organs, Guthrie on, 486
Seventy-second anniversary of medical society of London, 387
Shaw's medical Remembrancer, 499
Signs of auscultation in young children, Trousseau on, 49
Simpson, Prof., on unavoidable hæmorrhage, 367
Sixth annual report of the Ohio lunatic asylum, 220
Southern medical and surgical journal, 115
Society, ethnological, 445
" physico-medical, of N.Orleans, 564
" Sydenham, 347
Solly on mollities ossium, 44
Spencer's lectures on animal heat, 273
Spencer's, H. G. P., case of sewing needles and pins extracted from the wrist, 146
Spleen, enormous hypertrophy of, 580
" effects of extirpation of, 250
" alleged action of quinine on, 575
" new mode of examining, 575
Spontaneous rupture of the inferior cava, 57
Spontaneous rupture of the stomach, causing sudden death, 695
Spontaneous expulsion of the os hyoides, 196
Spine, Bamfield on curvatures and diseases of, 184
Squibb's, Dr. E. R., case of traumatic tetanus, 328
Squibb's, Dr. E. R., case of ovarian dropsy, &c., 457
Sternum, case of congenital deficiency, 60
Stewart's treatise on diseases of children, 663
St. Mary's, winter climate of, 335
Stomach and colon, cancerous communication of, 201
Stomach, spontaneous rupture of, 695
Stricture of the intestinal canal, 471
Strictures of the rectum, Mütter on, 77, 148
" " simple, 78
" " spasmodic, 78
" " permanent, 80
" " malignant, 145
" " treatment of, 152
Structure and diseases of the testis, Sir A. Cooper on, 109
Strychnia in chorea, 582
Sturgeon, Smith's experiments on the heart of, 393
Sudden blindness, case of, 200
Sudden death, cause in the brain, 630
" " with congestion of right heart and lungs, 632
" " from syncope, 634
" " from epilepsy, 635
Subcutaneous punctures, 700
Sulphate of quinine, toxical effects of, 377
" " Prof. T. D. Mitchell on, 386
" " alleged effects on the spleen, 575
" " not absorbed when applied endermically, 695
Surgery, report on the progress of, 736
Surgical memoranda by M. Vidal, 197
Surgical cases by M. Syme, 309
Surgical society of Ireland, 379
Surgery, Miller's principles of, 228
" general, Elliott's contributions to, 389
" orthopedic, Bigelow's novel application of electricty to, 441
" Colles's lectures on, 498
Sydenham society, 347
Syphilis eruption affecting the conjunctiva, 198
Syphilis, ioduret of potassium in, 562
Syrian medical association, 237

Tænia solium, escape of, by the umbilicus, 96
Testis, Sir Astley Cooper's observations on, 109
Tetanus, traumatic, intoxicating doses of alcohol in, 319
Tetanus, traumatic, Dr. Squibb's anomalous case of, 328

Therapeutics of Nature, the, 194
Therapeutics, 716
The seven books of Paulus Ægineta, 347
Thompson's domestic arrangement of the sick room, 622
Thymus gland, anatomy of the, 109
" " use of the, 196
Tobacco, Diver on the endermic use of, 527
Tongue, removal of part of the base of, 53
Toothache, caoutchouc in, 302
Toxical effects of sulphate of quinia, 377
" " of copper and zinc, 378
Tracheotomy in croup, 45
Traite d'hygiène publique et privée, par M. Levy, 541
Transactions of the college of physicians of Philadelphia, 704
Transylvania university and the navy, 419
Treatment of chancres, 54
" of hydrocele by ioduretted injections, 69
" syphilitic affections, 439
" and cause of chlorosis, 443
" of typhus, French and English opinions on, 509
Tremors, 270
Tumours, pulsating, of bone, 306
" malignant of inferior maxillary, 315
" fibrous of the inferior maxilla, 316
" aneurismal of bone, 375
" intra-thoracic, solid, 626
Turnbull's case of anasarca and ascites from pressure of a tumour, 592
Twins, one being black and the other white, Cunningham's case of, 647
Typhoid fever, Dr. Voigt's case of, 715

Ulceration of the duodenum, &c., 50
" of the rectum, 403
" simple of the os uteri, 449
" of the cervix uteri, 570
Ulcers, of the cornea, 555
University of Pennsylvania, report on the medical department of, for 1845, 621
Upshur's, Dr. G. L., remarks on iodide of potassium in pneumonia, 323
Urinary and sexual organs, Guthrie on, 486
Use of the Thymus gland, 196
Uterus, Dr. Mitchell's case of racemiferous hydatids of, 73
Uterus, anterior obliquity of, 573
Uretro-plastie, memoir on, 640
Utility of microscopical investigations, 572

Vagina, occlusion of in a pregnant woman, 255
Vaccination, French report on, 560
Valerianate of zinc, 361
Veins, occasional pulsation of, 194
Velpeau's new elements of operative surgery, 175
Velpeau's elements of midwifery, 607
Ventricles of heart, communication between, 261
Venous pulse on the back of the hand, 761
Veratria, adulteration of, with lime, 626
Vidal's surgical memoranda, 197
Voice, Colombat de l'Isere on the hygiène and diseases of, 357
Washington University of Baltimore, annual circular of, 62
Water's case of cancerous communication of stomach and colon, 201
Watson's lectures, 602
White swelling of knees, section of flexor tendons in the, 56
Williams's American medical biography, 110
Williams and Clymer on the respiratory organs, 103
Wells' hospital, 22
Winter climate of St. Mary's, 335
Women, Ashwell on diseases of, 225
Wood and Bache's dispensatory, 416
Worcester on cutaneous diseases, 350
Works of Hippocrates and Galen, 618
Worthington's case of fistulous communication between the ileum and bladder, 43
Wound, punctured, of abdomen, 643
" of the head with loss of substance of the brain, 696
Wounds, dressing of, by occlusion, 710

Yale College, medical institution of, 624
Yellow fever at Woodville, Miss., 117
" " post-mortem caloricity of, 624

Zinc, valerianate of, 361
" toxical effects of, 378
Zymotic diseases, 666

